# New Insights into the Role of Trace Elements in IBD

**DOI:** 10.1155/2018/1813047

**Published:** 2018-09-06

**Authors:** Georgiana-Emmanuela Gîlcă-Blanariu, Smaranda Diaconescu, Manuela Ciocoiu, Gabriela Ștefănescu

**Affiliations:** ^1^Department of Gastroenterology, Grigore T Popa University of Medicine and Pharmacy, Iași, Romania; ^2^Department of Pediatrics, Titu Maiorescu University, Faculty of Medicine, Bucharest, Romania; ^3^Department of Pathophysiology, Grigore T Popa University of Medicine and Pharmacy, Iași, Romania

## Abstract

Micronutrient deficiencies are common in inflammatory bowel disease and have clinical impact, being both a sign of complicated disease and a cause of morbidity. The involved systemic inflammatory response is responsible for altering the concentration of a wide range of trace elements in the serum, including zinc and selenium. This review summarizes recent advances and evidence-based knowledge regarding the impact of selenium and zinc on oxidative stress and microbiota changes in IBD patients. Getting new insight into the impact of malnutrition, particularly on the micronutrients' impact on the development, composition, and metabolism of microbiota, as well as the influence of oxidative stress and the mucosal immune response, could help in implementing new management strategies for IBD patients, with focus on a more integrated approach.

## 1. Background

Inflammatory bowel disease (IBD) has been the focus of basic science and translational-clinical research, resulting in the exponential growth of knowledge regarding its predisposing factors, possible cause(s), and underlying cellular and molecular mechanisms. To a large extent, this indisputable progress is due to a much improved understanding of IBD pathogenesis and the identification of its major components. Variations in the composition of gut microbiota and the reactivity of the intestinal mucosal immune response, along with dietary changes, have been extensively studied in the previous years in the pathogenesis of IBD. However, the majority of studies have eluded the integrative approach, so the knowledge acquired in one area does not efficiently translate and apply to the benefit of other components [1].

Among the consequences of dietary changes, restrictive diets, and absorption deficiencies, malnutrition, whether it only includes protein malnutrition or micronutrient deficiencies as well, is a frequent diagnosis that can persist further on, as a consequence of altered intake, use of various drugs, or hypercatabolic state in this patient category [2].

The systemic inflammatory response related to either acute or chronic inflammation is responsible for altering the concentration of a wide range of trace elements and vitamins in the serum. The presence of micronutrient deficiency is associated with a higher risk of poor outcomes, due to prolonged hospitalization [3], perioperative evolution, and growth deficit [4].

Among micronutrients, selenium (Se), particularly as a component of selenoproteins (mainly selenoproteins S and K), has been shown to impact the inflammatory signaling pathways involved in IBD pathogenesis, including the inflammatory cytokine production [5–7].

Zinc deficiency is common in patients with inflammatory bowel disease (IBD), during both active and remission phases, with a prevalence ranging from 15% to 40% [8, 9]. Studies on animal models and translational studies proved that decreased serum zinc concentrations may enhance inflammation through various pathophysiological mechanism, including disruption of epithelial barrier, altered mucosal immunity, and increased proinflammatory cytokines [10–12].

Most micronutrient status evaluations were performed by assessing serum levels [13, 14], although the presence of chronic inflammation determines a high variability among these, independently of tissue stores [15, 16]. Consequently, serum levels do not offer an accurate measure of trace element stores and trace element levels in hair have been proposed as a more reliable measurement of the chronic microelement nutritional status, considering that the systemic inflammatory response has been shown to independently decrease serum levels of micronutrients, including zinc, selenium, copper, and various vitamins, without any correlation to the actual nutritional status [2].

There is pathophysiological background for micronutrient deficit during inflammation, especially in the suppression of carrier proteins synthesis in the liver, due to proinflammatory cytokines; this leads to the sequestration of some trace elements in the liver as a consequence of the inflammatory response. This type of deficiency is common at diagnosis, obviously due to impaired absorption, but may also persist throughout the course of the disease due to various factors, such as poor intake in the context of restrictive diet, direct intestinal loss, or a hypercatabolic state in IBD patients. Given the significant role of zinc and selenium deficiency in determining poor outcome for IBD patients, which suggests that these micronutrients could be potential therapeutic candidates for IBD, this review will focus on various pathophysiological manners in which micronutrient deficiency could result in impaired evolution of IBD patients and how research into these mechanisms could impact IBD therapy.

## 2. Trace Elements Deficiency and Oxidative Stress in IBD

### 2.1. Inflammation and Oxidative and Nitrosative Stress (IO&NS) Pathways in IBD

Since the gut is a large interface with the environment, it is natural for it to be under high immune surveillance, including macrophages and important networks of dendritic cells, with important roles in adaptive immune responses [17]. Experimental studies on IBD have shown an increase in these immune cell populations together with an increased secretion of proinflammatory cytokines, of which IL-6, IL-13, IL-17, IL-22, and IL-23 proved to have an important share in the evolution of IBD. [18–23]. IBD is characterized not only by an increased immune-inflammatory response, but also by a reduced activity of suppressive cytokines TGF-*β* and IL-10 [24, 25]. There is also substantial evidence that the chronic inflammatory process in the intestine is closely related to oxidative and nitrosative stress, with impact on oxidative injury biomarkers, including lipid peroxidation products and protein changes in both UC and CD patients [26, 27]. The cellular sources of oxidative and nitrosative stress identified on animal models of IBD include macrophages and neutrophils, which generate important quantities of nitric oxide and superoxide [26]. The presence of proinflammatory cytokines enhances the production of NADPH-oxidase (NOX) and iNOS by the epithelial cells, consequently amplifying oxidative stress [28]. Several other reactive oxygen species (ROS) producing pathways are shown to be involved in the pathogenesis of IBD, such as xanthine oxidase, 5-lypoxigenase, and cytochrome P450 enzymes. The impact of prooxidative status is further accentuated by decreased antioxidant levels, which is also present during remission, suggesting that oxidative stress plays an important part in disease recurrence [26, 29]; this idea is further reinforced by the clinical efficacy of antioxidant therapy, such as melatonin, which is also a powerful anti-inflammatory and antioxidant [30].

Another hypothesis states that mitochondrial dysfunction could also be an important source of ROS, ergo the factors influencing mitochondrial functioning could enhance the inflammatory response by means of variations in terms of production or response to melatonin [31, 32].

### 2.2. Zinc Deficiency and Oxidative Stress in IBD

Zinc deficiency was recently shown to correlate with inflammatory status in IBD. A possible explanation for zinc playing an anti-inflammatory role in IBD could be related to its role in reducing the trans-mucosal leak in Crohn's disease, by decreasing the number of proinflammatory cells and reducing proinflammatory cytokine production [33, 34].

Zinc is a trace element known for its role in cell turnover and repair systems, with studies showing that correcting zinc deficiency can lead to restoring intestinal permeability in CD patients, probably due to its ability to modulate tight junctions both in the small and the large bowel [35].

In terms of immunity, zinc is essential for cell proliferation and influences both the acquired and innate immunity by also acting as a coenzyme in many key reactions of the immune response, being essential for antioxidant response and thymic hormone function [36]. Zinc deficiency leads to impairing or even completely suppressing the phagocyte and lymphocyte activity, determining an inefficient cytokine response [37–39]. Moreover, it has been reported that, in activated macrophages, zinc, as a component thereof, suppresses the activity of inducible nitric oxide synthase (iNOS) by about 90%, preventing the production of reactive oxygen and nitrogen species and cellular damage [36]. Together with zinc, copper (Cu) plays a more important role among micronutrients in terms of inflammation, with increased Cu/Zn ratios being identified in cases of chronic inflammation and free radical overproduction [40], although serum Cu concentrations were inconsistent across various studies on IBD patients compared to healthy controls [36, 40, 41]. The differences registered between the studies may in part be due to various degrees of disease activity. However, one study showed significant serum Cu elevation in women with IBD compared to healthy subjects [36].

The Cu/Zn-SOD is considered the main isoform active in IBD and its expression had been previously reported [42]. Various studies confirmed the reduction of Cu/Zn-SOD activity in IBD patients, which means a reduced ability to scavenge free radicals in IBD patients. The reduction of this SOD isoform may be in part due to Zn deficiency and partly due to chronic inflammation, but the underlying mechanisms are yet to be clarified [36, 43].

Moreover, a study on* in vitro* cell culture has shown that zinc deficiency leads to increased interleukin1b and interleukin-6 responses following lipopolysaccharide stimulation, underlining a potential pathway between microbiota components with immune response and micronutrient deficiency [44].

Considering the role of Zn in offsetting oxidative stress, Zn supplementation has been considered in IBD patients. However, one study highlighted that although zinc supplementation, in the form of zinc gluconate, improves the homeostatic condition of this trace element, it did not change SOD activity, as a marker of oxidative stress in patients with ulcerative colitis [45].

In the pursuit to use zinc as part of the therapeutic approach to IBD, another strategy applied in experimental studies was to incorporate Zn in cross-linked blend microspheres; this used Zn for its dual effect, both as a cross-linker to form drug delivery carriers in colon-specific drug delivery systems and for its anti-inflammatory role, delivered together with 5-aminosalycilate derivates (5-ASA), which proved to alleviate colonic inflammation and promote mucosal healing in a mouse model of TNBS-induced colitis. Consequently, Zn ion cross-linked alginate/N-succinyl-chitosan blend microspheres could emerge as suitable candidates for the codelivery of zinc and 5-ASA into the colon, with potential therapeutic effects in IBD [46].

In the attempt to counteract the oxidative stress by excessively scavenging generated ROS, the efficacy of zinc in the form of zinc oxide nanoparticles (ZnO np) has been investigated on an animal model of ulcerative colitis. Using a model of DSS-induced colitis, there is evidence to the antioxidant and anti-inflammatory abilities of ZnOnps in suppressing ROS and malondialdehyde (MDA) production, increasing GSH levels, and suppressing proinflammatory cytokines IL-1*β* and TNF-*α* and myeloperoxidase. The proposed mechanism is the activation of the Nrf2 pathway in the cellular antioxidant defense system. The novel finding of this study using a DSS-induced colitis model is the synergic potential of ZnOnp with mesalazine, with greater therapeutic efficacy than mesalazine alone, and also the ability of ZnOnp to restore the colonic microbiota of the DSS-mice, while mesalazine alone cannot [47].

### 2.3. Selenium and Oxidative Stress in IBD

Another micronutrient and trace element with involvement in antioxidant response is selenium (Se), exerting its biological effects through selenocysteine, an amino acid which is incorporated into proteins [48]. Se deficiency has been recorded in both UC and CD patients, and its deficit was correlated to an increased risk for multiple chronic inflammatory conditions, such as cardiovascular or endocrinological (thyroid) disease [49].

Se deficiency was found to occur in IBD patients even during remission, and consistently low Se serum levels were associated with increased severity of the UC and CD activity index, even suggesting that Se might become a noninvasive biomarker for IBD activity and severity [50, 51].

In this context, this trace element is particularly important due to its incorporation in selenoproteins with potential to modulate local inflammatory response. Among selenoproteins, the most intensively studied for their role in ROS reduction were the four isoforms of glutathione peroxidases (GPx), which are expressed in the gut [5]. While GPx1 is expressed by all intestinal cell types, GPx3 is secreted and found in plasma. GPx2 is mainly expressed in the epithelial cells, including the Paneth cells, and GPx4 is found both in intestinal epithelial cells and in the lamina propria [52]. GPx isoforms GPx1 and GPx2 are differentially regulated, with GPx2 being upregulated as part of a compensatory response for protection against oxidative damage during inflammation [53]. However, there is evidence on animal models of GPx2 knock-out mice, which were fed with Se, that there is increased GPx1 activity within the colon and ileum crypts, supporting the hypothesis that these proteins may have a partial compensatory role [54]. Furthermore, the absence of both GPx1 and GPx2 leads to a severe inflammatory response, exhibited as spontaneous ileocolitis [55]. As for the other isoforms with antioxidant role in the gut, the loss of GPx3 was linked to inducing severe colitis, while plasma GPx3 showed potent tumor suppressor role in colitis-associated carcinoma through abrogation of ROS [56]. GPx4 has an important role in reducing lipid peroxidation, consequently preventing membrane disruption due to oxidative stress and helping maintain cellular integrity. Although GPx4 is uniformly expressed in the colon, its expression is variable throughout the crypt-villus axis [57].

Except for GPx4, there is another quite similar selenoprotein, but with significantly lower activity, namely, selenoprotein P. This is one of the major plasmatic selenoproteins, with a high sensitivity to changes in serum Se level, considering its high content of Sec residues and its role in antioxidant response, mainly responsible for delivering selenium to different tissues [58]. However, its levels are inversely associated with the development of IBD, which may be due to the association of low Se absorption and IBD development; another related aspect might be the fact that low plasma levels of selenoprotein P impact selenoprotein expression in target cells such as macrophages [52, 59].

Another type of selenoprotein, selenoprotein S (seleno S), highly expressed in Paneth cells and macrophages in the gut, is a marker of endoplasmic reticulum stress at this level but does not regulate the process. Seleno S is thought to have two main roles: supplying tissues with Se and acting in the antioxidant defense network. In the context of IBD, increased production of inflammatory cytokines has been reported concurrently with a decrease in the expression of several selenoproteins, including seleno S [60]. Studies on animal models have reported that Se in the form of selenoproteins can influence macrophage activity, by preventing the arachidonic acid pathway to generate proinflammatory mediators such as PGE2 and preventing interleukin- (IL-) 1 to generate more anti-inflammatory mediators such as prostaglandin D2 (PGD2) and some of its metabolites [61]. The reduction of Se plasma level was also shown to be correlated with increased plasma level of prostaglandin E2 (PGE2) in ulcerative colitis patients, an increase in prostaglandin E2 (PGE2) in the plasma of patients with ulcerative colitis, therefore with a proinflammatory response [62]. Interestingly, an indirect proinflammatory effect has been reported for selenoprotein K, its absence determining a decrease in inflammatory cytokines [6]. These changes are context-dependent and require further research.

Furthermore, a few experimental studies using models with DSS-induced colitis and associated colon cancer suggest that Se and selenoproteins play an essential role in regulating inflammatory microenvironment and tumorigenesis [63, 64]. In addition, experimental studies have also demonstrated that there is reduction of cytokines known for their proinflammatory effect, such as IL-1, TNF*α*, and IFN*γ* and concomitant increased level of anti-inflammatory markers expression, including arginase 1 proinflammatory cytokines such as IL-1, tumor necrosis factor alpha (TNFalpha) and interferon gamma (IFNgamma), and increased anti-inflammatory markers such as arginase 1, secondary to Se supplementation in mice with DSS-induced colitis [65, 66].

Regarding the relevance of effector cells in oxidative stress, the M2 type macrophages, the alternatively activated macrophage subtype, are of particular interest [67]. In contrast to M1 macrophages, which are considered proinflammatory due to their involvement in ROS production and activation by tumor necrosis factor- (TNF-) *α*, lipopolysaccharide, and other Toll-like receptor (TLR) ligands [68], M2 macrophages are considered anti-inflammatory due to their increased expression of arginase-1, which competes for l-arginine; L-arginine is a substrate for inducible nitric oxide synthase, diverting nitric oxide synthesis and leading to the production of l-ornithine and urea instead [69]. The role of Se supplementation in switching from M1 to M2 type, ergo from pro- to anti-inflammatory status, could reside in the epigenetic changes [52]. Kudva et al. demonstrated the ability of Se, dependent on the selenoprotein-mediated shunting of arachidonic acid pathway, to inhibit the acetylation of nonhistone and histone proteins and therefore to affect the expression of proinflammatory genes in macrophages, including NF-*κ*B member p65 [52, 70, 71]. Therefore, Se (through selenoproteins) seems to effectively shunt the eicosanoid pathway, driving the production of PGD_2_ and its metabolites that potentially influence NF-*κ*B- and PPAR*γ*-dependent pathways, thus constituting one of its many anti-inflammatory functions [52, 72].

Although the influence of Se and PPAR*γ* on the evolution of IBD and the mechanism of Se impact on oxidative stress in this context have not represented the focus of many studies, to our knowledge, there has not been a research core on the effect of Se and PPAR*γ* on IBD and, based on the available data, one could infer, using murine models of IBD [73, 74], that given the reduction in PPAR*γ* in IBD, as well as the potential of Se to enhance PPAR*γ* and its ligand 15d-PGJ2 [75], Se supplementation would significantly decrease disease activity. This outcome could be interceded: it is plausible that under supplemented Se status the disease activity would be significantly decreased. This effect could be mediated through several pathways, including the upregulation of PPAR*γ*, which acts as inhibitor of NF-*κ*B activation in various cell types, such as intestinal epithelial cells, macrophages, and dendritic cells, influencing the production of proinflammatory cytokines, involved in the pathology of IBD [66]. Moreover, PPAR*γ* has been shown to regulate T cell activation [76] by inhibiting the differentiation of Th1 cells or the potential of these cells to produce cytokines.

In this context, it is important to note that Tregs expression during IBD is heterogenous and that PPAR*γ* has been shown to lead to an increased expression of Foxp3+ Tregs. This suggests that in IBD this could lead to an increased number of FoxP3+ Tregs in the colon, although Tregs are not the only T cells expressing FoxP3. Through the proven effect of selenium in activating PPAR*γ*, Se supplementation could exert its effects by inhibiting certain pathways or immune cell functions, which could lead to the active resolution of inflammation in the gut.

Therefore, several potential beneficial effects of Se supplementation become apparent. Various types of dietary and supplemental Se have been studied. However, using animal models leads to obtaining conflicting results in terms of Se supplementation under various forms (sodium selenite, selenomethionine) [77–79]. Se nanoparticles (SeNPs) seem to be more effective than other forms of Se in scavenging free radicals and therefore in preventing DNA oxidative damage, having low toxicity levels and acceptable bioavailability [80, 81]. There have been attempts to use SeNP directed into the gut mucosa for the treatment of IBD, mainly for local (rectal) use, due to their low adverse effects [82, 83].

Continuing these studies, Zu et al. found that there are several advantages to capping SeNP with agents such as ATP and vitamin C, such as enhanced cellular uptake, prolonged circulation of SeNP, as well as advantages related to the size and stability of SeNPs [84]. It is difficult to state whether Se supplementation is a feasible component of IBD therapeutic strategy and this debate requires further research. [52].

## 3. Microbiota and Trace Elements

There have been extensive studies on microbiota changes in IBD, suggesting that the relationship between dysbiosis and IBD is complex and dynamic, certainly not limited to the cause-effect relationship type. Consequently, assuming that dysbiosis is the response of a complex microbial community to the environmental inflammatory stress or medication might overlap with the hypothesis that it plays a direct role in IBD pathogenesis [85–87]. A microbiota imbalance might not actually be among the events involved in triggering inflammation, but it could develop later in the course of IBD and contribute to disease progression and chronicity; alternatively, dysbiosis could play a critical role in disease onset, but the window for such an effect occurs early in life. These aspects could be supported by studies of more targeted probiotics and by larger controlled trials on the use of fecal microbiota transplantation, supporting this hypothesis [85].

Since the composition of the gut microbiota can be altered by various exogenous factors such as infections, antibiotics, and diet, micronutrient deficiency could also play a part in affecting the gut mucosal immune response. It has already been stated that metal availability is among the critical factors influencing the outcome of host-microbe interactions [88]. Diet is a factor that is very likely to play a major role in metal availability, particularly during infections. Altered dietary microelement levels are associated with increased susceptibility to various infections; nonetheless, there is scarce data on how altered dietary metal levels affect the gut microbiome [89].

A previous report demonstrated that the composition of gut microbiota in mice affected host Se levels and therefore the selenoprotein expression in the host [90]. There is likely to be a competition between intestinal microbiota and the host for available selenium, exacerbating host selenium deficiency and increasing the vulnerability of the gut to disease. On the other hand, Se levels were shown to also alter the composition of gut microbiota in mice [73]. One aspect of particular interest would be studying the underlying mechanisms of Se influence on dysbiosis and whether this trace element deficiency correlates with incidences of IBD, modifying disease severity. Furthermore, in the attempt to translate basic research into clinical application, it would be useful to see whether there is a protective effect of Se supplementation mediated via microbial metabolite(s), not only by generating species selection, but also by assisting in diminishing inflammation or enhancing mucosal healing by modulating the host's immune response [52].

*Clostridium difficile* (CDiff) was studied among particular bacterial influence, also due to its high prevalence among IBD patients. The preliminary steps of CDiff infection include loss of colonization resistance and development of susceptibility, usually mediated by use of antimicrobial therapy, altering the gut microbiota and generating the respective susceptibility. On top of CDiff's virulence factors, host characteristics also play a role in this process. There are distinct risk factors for CDiff infection in IBD patients, including younger age, outpatient care, and lack of antibiotic exposure immediately preceding CDiff infection onset [74]. One study on a mouse model reported that excess dietary Zn exacerbates CDiff-associated disease, by decreasing the threshold of antibiotics needed to confer susceptibility thereto; this was shown using CFU analysis for the CDiff strain R20291 following low-level cefoperazone treatment [91].

Due to the anti-inflammatory effect [92] and the established low richness of mucosa-associated* Faecalibacterium prausnitzii *(FP) in IBD [93], its role in the evolution of this disease has also been studied. On animal models and using FP strains, its potent anti-inflammatory role was shown to be exerted via interaction with various immune pathways, by means of inhibiting IL-17 [94], inducing anti-inflammatory cytokines like IL-10 in dendritic cells [95], influencing Th17 differentiation [92], as well as by butyrate production and consequent inhibition of NF-*κ*B activation, leading to the activation of different genes involved in enterocyte differentiation, proliferation, and regeneration [96].

SeNP proved to increase SCFA production and FP abundance, with current major efforts underway to produce FP probiotics for the treatment of colitis. The increase rate in FP achieved using animal models, supplemented with nanoSe, exceeded expected levels of enrichment in the gut via orally delivered probiotic. This warrants further investigation in the use of nanoSe to enrich FP in order to improve both animal and human intestinal conditions using higher sample sizes and multiple trials. These results also encourage further investigation into optimal nanoSe concentrations for obtaining other benefits, which may have resulted from increases in SCFAs, such as reduced intestinal permeability and integrity [97]. Nevertheless, there may be difficulties in adding FP and including it in nanoparticles on the Qualified Presumption of Safety list due to no current information of safe use; furthermore, toxicological assays are required for regulatory approval [96, 98]. This might be difficult due to its lack of a history of safe use; moreover, full toxicology assays and characterization of the strain are still needed for regulatory approval [96, 98].

Based on our current knowledge, it is difficult to state whether there is a bilateral influence/link between the deficit of trace elements, especially regarding Se deficit and dysbiosis [73]. Some studies point to the use of some common immunological pathways, including regulation of both NF-*κ*B and PPAR*γ*, since commensal microbiota, which can be involved in IBD pathogenesis in the presence of epithelial barrier dysfunction and increased intestinal permeability and may also influence the activation of and can also regulate the activation of both pathways [99, 100]; consequently, further studies are required to clarify this aspect.

## 4. Conclusions and Future Directions

Selenium and zinc are essential micronutrients with a variety of roles in mediating immune response. Although IBD pathophysiology is multifactorial in origin, dietary zinc and selenium deficiency exacerbates experimental colitis by affecting various signaling pathways involved in inflammation and oxidative stress, as well as by altering the gut microbiota. For Se, this may be partially due to its ability to change macrophage phenotype, from a M1- to M2-dominant expression, therefore alleviating inflammation and reducing intestinal epithelial damage. Among the various cellular pathways, the ability of selenoproteins to lead to eicosanoid pathway shunting contributes to decreasing inflammation along with enhanced wound healing [52, 65]. Some authors reported that its form of administration and the duration of supplemental therapy may be significant for imparting the beneficial effects; however, since results are inconsistent, further research in this direction is required [77]. Considering the potential activation of NF-*κ*B and PPAR*γ* through both the action of Se at cellular level and some commensal bacteria with potential involvement in IBD pathogenesis, this trace element has an emerging role in activating several cellular types, including macrophages, Th1, Th17, as well as in microbiota modulation, with potential therapeutic approach [73, 100]. Zinc deficiency is related at least to altering phagocyte activity, by suppressing antioxidant response and lymphocyte activity, consequently disrupting cytokine response [36]. A question that remains highly debated is whether changes in Se and Zn concentrations are among the causes or effects of IBD. Therefore, the cause and effect relationship between trace elements, dysbiosis, and IBD requires further examination through the development of appropriate animal models, considering a broader variety of factors (including changes in microbiota and environmental factors such as diet), in addition to understanding the molecular basis of inflammatory response modulation, in order to identify new therapeutic approaches. Such studies may ultimately provide a solid foundation and better biomarkers to identify patient populations that could benefit from micronutrient supplementation therapy, as well as from the new generation of probiotics using nanoparticles.
